# Patient perspectives on current and potential therapies and clinical trial approaches for cocaine use disorder

**DOI:** 10.3389/fpsyt.2024.1230699

**Published:** 2024-02-29

**Authors:** Suzanne Maahs, Denise Leclair, Baltazar Gomez-Mancilla, Brian D. Kiluk, Velusamy Shanmuganathan Muthusamy, Partha S. Banerjee, Shyamashree Dasgupta, Katherine M. Waye

**Affiliations:** ^1^ Novartis Biomedical Research, Cambridge, MA, United States; ^2^ Novartis Pharmaceuticals Corporation, East Hanover, NJ, United States; ^3^ Novartis Biomedical Research, Neuroscience Research, Basel, Switzerland; ^4^ Department of Neurology and Neurosurgery, McGill University, Montreal, QC, Canada; ^5^ Yale School of Medicine, New Haven, CT, United States; ^6^ Novartis Healthcare Pvt. Ltd., Hyderabad, Telangana, India

**Keywords:** cocaine use disorder, online bulletin board, individuals with current cocaine use disorder, individuals in cocaine use disorder remission, unmet needs, clinical trial outcomes

## Abstract

**Background:**

Cocaine use disorder (CUD) is characterized by the continued use of cocaine despite serious impacts on life. This study focused on understanding the perspective of individuals with current CUD, individuals in CUD remission, and their supporters regarding current therapies, future therapies, and views on clinical trials for CUD.

**Methods:**

The online bulletin board (OBB) is a qualitative tool where participants engage in an interactive discussion on a virtual forum. Following completion of a screening questionnaire to determine eligibility, individuals in CUD remission and their supporters logged in to the OBB and responded to questions posed by the moderator. Individuals with current CUD participated in a one-time virtual focus group.

**Results:**

All individuals with current CUD and 94% of those in CUD remission reported a diagnosis consistent with CUD or substance use disorder during screening. Individuals with current CUD and their supporters were recruited from the United States (US). Individuals in CUD remission were recruited from five countries, including the US. Individuals with current CUD reported hesitation about seeking treatment due to stigma, a lack of privacy, and being labeled as a drug seeker; barriers to therapy included time, cost, and a lack of privacy. Participants wanted a safe therapy to stop cravings and withdrawal symptoms. Seven clinical trial outcomes, including long-term abstinence and craving control, were suggested based on collected insights.

**Conclusion:**

This study can help inform the design of clinical trials and emphasize the need for effective, safe, and accessible therapies. Recruiting participants will require significant trust building.

## 1 Introduction

Cocaine is used by an estimated 20 million people globally, with an annual prevalence of use of 0.4% among those aged 15–64 years (1). Some of these individuals will develop cocaine use disorder (CUD), which is characterized by meeting at least two Diagnostic and Statistical Manual of Mental Disorders-5 (DSM-5) criteria such as “hazardous use”, “social or interpersonal problems related to use”, and “neglected major roles due to use” (2). The 2016 Global Burden of Disease study estimated an age-standardized prevalence of 77.6 CUD cases per 100, 000 people worldwide (3). Furthermore, an increase of 39.7% in CUD diagnoses was reported from 2010 to 2016 (4), and rates of overdoses, including cocaine overdoses, have recently increased exponentially (5, 6).

Although there are approved pharmacotherapies for opioid, tobacco, and alcohol use disorders, the treatment options are limited for CUD, with no approved pharmacotherapies in the United States (US) or Europe despite multiple clinical trials in the last several decades (4, 7, 8). Brain stimulation modalities such as deep brain stimulation (DBS) and repetitive transcranial magnetic stimulation (rTMS) are add-on therapies that have an acute effect on a person’s craving for drugs and alcohol. These modalities are utilized when pharmacological interventions are not successful, but may not always be available in certain regions or considered cost effective (9). Even though psychosocial and behavioral interventions have demonstrated efficacy in CUD, there is no one superior treatment, resulting in the use of a range of therapies (10). These therapies include evidence-based approaches such as individual counseling, cognitive behavioral therapy (CBT), community reinforcement approach (CRA), and contingency management (CM) (8). These treatments often demonstrate clinical benefit, but long-term abstinence remains elusive for the majority of patients with CUD (7–9, 11).

Clinical trials frequently consider abstinence from cocaine use as the criterion of therapy efficacy (12). However, abstinence can be a difficult outcome to achieve, and may not be the ultimate goal for some patients. Recently, the US Food and Drug Administration issued a status update declaring their interest in the use of novel, non-abstinence-based outcomes as part of product development, along with the development of new products that address a fuller range of symptoms of addiction, such as cravings (13–15).

Clinical trial participation and the response of the participant to psychotherapy are interconnected. Relapse rates are high in substance use disorder (SUD) studies, and dropout is an important predictor of relapse (16). Approximately 30% of participants drop out of SUD trials, and studies targeting cocaine use are associated with higher dropout rates than those involving tobacco smoking and heroin use. Because of this high dropout rate in SUD trials, it is difficult to observe the beneficial effect of treatment.

Supporters (defined here as friends or family members involved in caring for a patient with CUD) also play an important role in the effective treatment of patients with CUD. However, owing to the social stigma associated with CUD, few supporters share their burdens and perspectives, or receive support from their community (17). Some studies have explored the burden of the supporters in taking care of loved ones with alcohol use disorder (18–22) and with SUD (17, 22–24), but, to date, no studies have provided the supporters’ perspectives on CUD and treatment options for the individuals they care for and support.

The online bulletin board (OBB) method used in this study is a moderated, qualitative tool where participants engage in an interactive discussion in an open virtual forum (25). To date, studies using an OBB have provided valuable insights into different health conditions (25–28). To better understand patient and supporter perspectives, the current study used both an OBB and a supplemental virtual focus group to try and determine (1) patient and supporter preferences on an ideal therapy for CUD (2), patient and supporter perspectives on the design and execution of future CUD clinical trials, and (3) clinical trial potential outcomes that are the most important and useful to patients with CUD.

## 2 Methodology

### 2.1 Study design and setting

This study was conducted over 2 weeks from February to March 2021 across the following groups: individuals in CUD remission (five OBBs in the US, United Kingdom, France, Spain, and Brazil with individuals who reported they had recovered from CUD); supporters (one OBB in the US with friends or family members of someone with CUD); and individuals with current CUD (one focus group in the US with individuals currently diagnosed with CUD). An external vendor specializing in market research methodologies partnered with BGM, DL, SM, and KW to lead all recruitment, screening, and moderating efforts for the OBB and focus group.

A mix of recruitment methods were used, including contacting participants on market research panels, social media advertising in relevant special interest groups, and referrals from volunteers at rehabilitation clinics. Interested participants would then complete a series of screening questions (18 to 22 questions) over a secure survey platform. They would then receive a follow-up phone call from a trained staff member to confirm their eligibility and schedule their time in the OBB or virtual focus group.

Individuals in CUD remission and their supporters were requested to log in to the OBB for a total of 60 min split across 8 days over a 2-week period and respond to the questions posed by a trained moderator that spoke in the participant’s respective native language. Depending on the day of the OBB, participants were asked anywhere from a total of 5 to 13 questions (**Supplementary Material 2: Screeners**). Participants were encouraged to engage in anonymous discussions with other participants to exchange their views and experiences.

Individuals with current CUD were requested to join a virtual focus group once for a 90-min discussion that was conducted by a trained moderator who asked a series of 40 open-ended questions that explored the objectives in Section 2.3. The virtual focus group occurred on a confidential online platform (InterVu, FocusVision, USA) to ensure that participant names and/or locations were not displayed. All recordings of the focus group were destroyed after transcription.

For the purpose of simplicity, pharmacological treatments, 12-step programs, and other community mutual help groups were included within the definition of therapies, as many individuals with SUD engage in these programs as an integral part of their remission.

### 2.2 Participants

During screening, to qualify as an individual with current CUD, participants had to report the use of “cocaine” or “crack cocaine” regularly, report a diagnosis consistent with CUD or SUD, or answer “yes” to at least five DSM-5 criteria questions about CUD to ensure the inclusion of patients with self-reported CUD to have moderate to severe CUD. Individuals in CUD remission must have said “yes” to regularly using “cocaine” or “crack cocaine” in the past, reported a past history of substance use—including cocaine use—or must have said “yes” to at least five DSM-5 criteria questions to attempt to ensure the inclusion of moderate to severe CUD.

A supporter was defined as a friend or family member of someone who regularly used cocaine. A supporter was eligible to participate if they stated they were caring for or supporting someone currently using “crack cocaine” or “cocaine”. They must have said “yes” to five or more DSM-5 criteria questions about their loved one’s current cocaine use or confirmed a past diagnosis of CUD or SUD.

### 2.3 CUD OBB and focus group study objectives

The study objectives were as follows:

a) Gather participants’ insights into current therapies.
b) Assess satisfaction levels with current therapies.
c) Identify barriers to accessing treatment.
d) Obtain participants’ insights into an ideal future therapy for CUD.
e) Obtain participants’ insights into the design of future clinical trials for CUD therapy.

### 2.4 Qualitative analysis of participant responses

The main output for the OBB and the virtual focus group was a transcript of the discussion. The OBB also generated some additional outputs, such as images and responses to closed questions (e.g., agreement with statements or polling) that were analyzed alongside the transcripts. The anonymized data underwent thematic analysis by trained staff. Transcripts were reviewed iteratively by several team members to identify and examine key themes and patterns within interviews, within and across markets and patient demographics.

Additional details on the methodology as well as screener, OBB, and focus group questions can be found in the **Supplementary Material Sections 1**-**3**.

## 3 Results

### 3.1 Participants

All individuals with current CUD (*N* = 5) and 94% of those in remission reported a diagnosis consistent with current or past CUD or SUD. The remaining 6% of those in remission reported cocaine use consistent with CUD by patient history and DSM-5 criteria. Individuals with current CUD and supporters participating in the study were aged 22–49 years (*n* = 5) and 33–53 years (*n* = 6), respectively. Those in CUD remission (*N* = 35) were aged 22–65 years and were from Brazil (*n* = 7), France (*n* = 4), Spain (*n* = 6), US (*n* = 12), and UK (*n* = 6). Details of participant demographics are presented in **Table 1**.

**Table 1:** Demographics of individuals with current CUD, individuals in CUD remission, and supporters.

Individuals with current CUD					
Country			US		
Age range			22–49 years		
Sex			Male, *n* = 5		
Individuals in CUD remission					
Country	Brazil	France	Spain	UK	US
Age range	30–60 years	23–50 years	30–60 years	30–49 years	22–65 years
Sex	Female, *n* = 2	Female, *n* = 2	Female, *n* = 1	Female, *n* = 4	Female, *n* = 9
	Male, *n* = 5	Male, *n* = 2	Male, *n* = 5	Male, *n* = 2	Male, *n* = 3
Time in remission (range)	1 year–24 years	2 months–10 years	3 years–18 years	1 month–14 years	35 days–10 years
	•	•	•	•	•
Supporters					
Country			US		
Age range			33–53 years		
Sex			Female, *n* = 6		
Relationship with CUD participant			Aunt, cousin, sister, friend, spouse, parent		
Living with CUD participant			Yes, *n* = 5		
CBT, cognitive behavioral therapy; CUD, cocaine use disorder; UK, United Kingdom; US, United States.					

### 3.2 Insights from participants regarding current therapies and barriers to treatment

#### 3.2.1 Individuals with current CUD

Individuals with current CUD reported moderate satisfaction with available therapies (average score of 4; scale of 1–7, with 7 being the highest possible score). Residential rehabilitation and 12-step programs prompted some negative feedback, while counseling and CBT were preferred. These individuals felt that society considered them as “once an addict, always an addict,” and that admitting they had a problem to a peer support group was challenging. Off-label medications received mixed views, as there were concerns about dependence and having to go to a healthcare professional (HCP). Most considered seeking professional help but were distrustful of HCPs due to concerns about a lack of anonymity or of being judged negatively by the HCP. Those who had approached HCPs were worried about the interventions being documented permanently in their medical records and being perceived as drug seekers in the future. Having a good connection with an HCP was considered a crucial factor for remission (**Supplementary Material 4; Table S1**).

Lifestyle changes were appealing to individuals with current CUD who were not interested in receiving formal treatment.

This participant group listed several barriers to available therapies, such as the time and effort required, the cost, and skepticism about the effectiveness of therapy. For working professionals, a concern was the potential stigma of requesting medical leave for the treatment of CUD. A summary of current therapies and barriers can be found in **Table 2**.

**Table 2:** Insights from individuals with current CUD and supporters regarding current therapies/community mutual help groups used by CUD patients.

Preferred therapies for individuals with current CUD										
Counseling	Lifestyle changes	Peer support	12-step program	CBT	Individual/family therapy	Residential rehabilitation	Medication	Alternative medication	Mobile applications	Medical devices
High appeal	Low appeal	No appeal	Negative reaction	High appeal	No mention	Negative reaction	Mixed reaction	Low appeal	Low appeal	No mention
• Provides a level of accountability • Lack of judgment • Direct feedback • The biggest drawback is cost	• Relevant only to self-reliant users	NA	• “Once an addict, always an addict” • Seen as an outdated method with religious links • Can introduce users to peers to then take drugs with	• A practical option that allows users to learn how to change their behavior • One participant had tried this but with no long-term success • The biggest drawback is cost	NA	• Immersive but not practical • Expensive • Has a logistical impact on employment • Isolated from triggers and as such does not mimic real life	• Interested in potential new options • Concerns around anonymity • Would have to speak to HCP to access • Could become addicted to medication	• Interest was shown by a few participants	• Appealed to those looking toward taking practical steps	NA
Preferred therapies by supporters										
High appeal	No appeal	No appeal	No appeal	No appeal	No appeal	Supporters looking for total environment change	No appeal	Low appeal	No appeal	No appeal
Barriers to treatment for individuals with current CUD										
Time		Stigma		Cost		Hard work		Skepticism		
The time required for therapy is one of the biggest barriers, with respondents reluctant to try something that will take a chunk of time from their life		Respondents are keen to hide their addiction from employers and as such cannot take much time off, and are hesitant for anything to be on their medical records		Money is a huge barrier; many of the treatments mentioned by respondents are highlighted as costly and difficult to access		A number of therapies are thought of as “hard work” and can initially be off-putting for respondents, particularly those who do not think they have an addiction problem and believe they can quit on their own		Respondents are skeptical about therapies; many therapies are seen as an expensive waste of both time and money with poor success rates, and as such, not something they feel particularly enthused about		

CBT, cognitive behavioral therapy; CUD, cocaine use disorder; HCP, healthcare professional; NA, not applicable.

#### 3.2.2 Individuals in CUD remission

**Figure 1:**
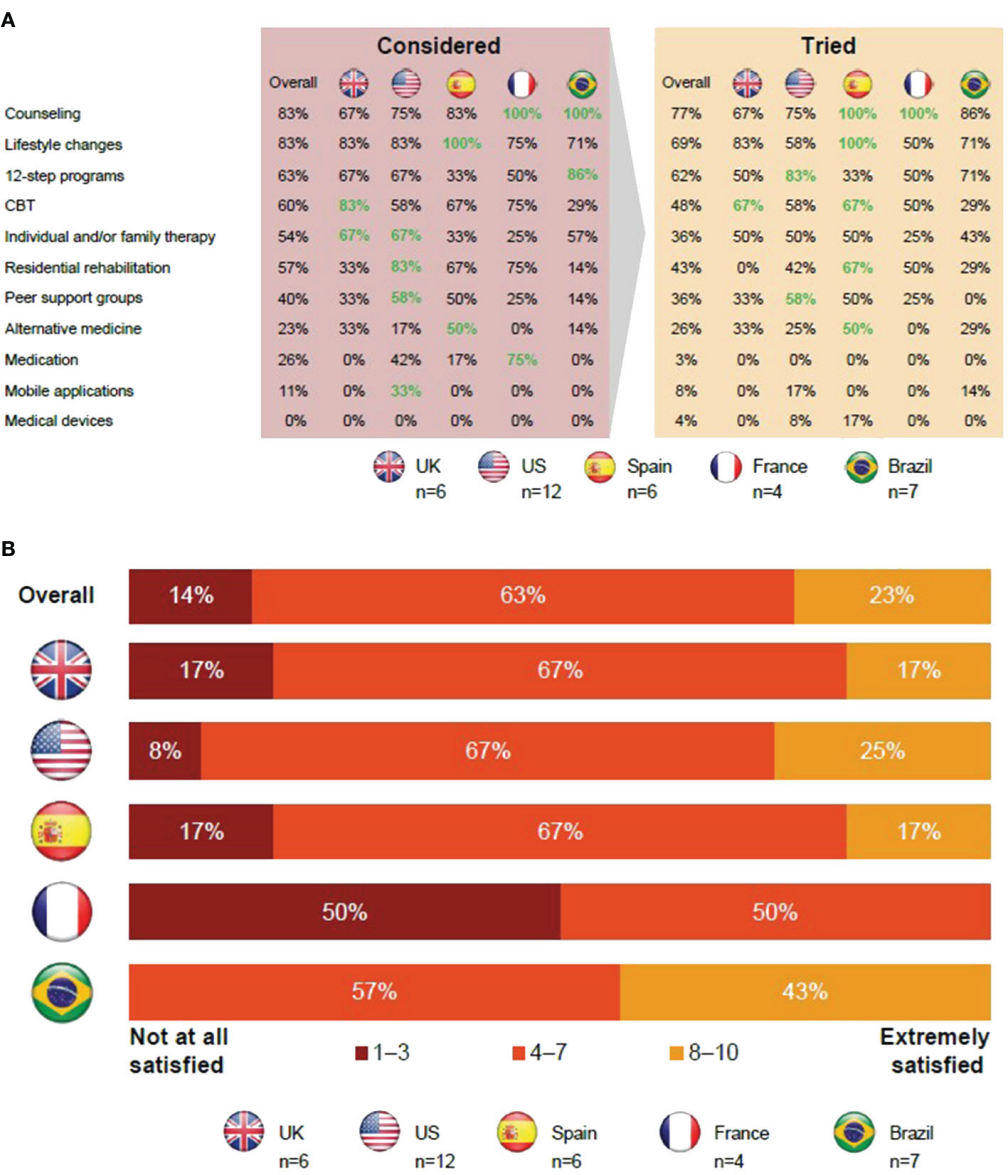
Insights from individuals in CUD remission regarding current therapies for CUD. **(A)** Those in CUD remission favored 12-step programs and cognitive behavioral therapy. **(B)** Most individuals in CUD remission were dissatisfied with current therapies. Apps, applications; CBT, cognitive behavioral therapy; CUD, cocaine use disorder; UK, United Kingdom; US, United States.

Of those in CUD remission, 86% were satisfied with the available therapies for CUD (**Figures 1A, B**). Individuals in CUD remission thought that CUD was not taken as seriously as other addictions, such as alcohol or opioid use disorders, so there were fewer options available (**Supplementary Material 4; Table S1**).

Overall, 77% of those in CUD remission tried counseling as a form of treatment, with all participants from Brazil and France attempting it. A majority of the participants highlighted the neutral, judgment-free, professional approach to listening and being provided with useful guidance as the reason for trying counseling. However, either the perceived high cost or lack of health insurance coverage was a barrier for accessing counseling.

The next most commonly tried approaches were lifestyle changes and 12-step programs with 69% and 62% of participants having tried them, respectively. These approaches were reported to have few cost-related barriers; however, participants believed that it was critically important that individuals had sufficient motivation to try these approaches. Peer support groups and 12-step programs were favored by older (aged >49 years) individuals in CUD remission.

The majority of participants considered engaging in CBT, and 48% actually tried it.

In the UK, CBT is available through the National Health Service and can also be free or compulsory if compelled by law enforcement. However, in other locations, participants reported that it may be more difficult to access or find CBT options.

Individual and/or family therapy was tried by 36% of those in CUD remission. Participants thought therapy could help them recover from CUD and address other factors that led to their CUD. Involving family in therapy was viewed as a chance to mend relationships and allows the participant’s family to better understand them. However, participants did state that involving family can be uncomfortable.

For participants in the US, residential rehabilitation was widely known, but the quality of such residential facilities was thought to vary in quality and approach. Residential rehabilitation was favored by those aged <49 years but was also deemed expensive and there was a perceived risk of relapse upon returning to everyday life.

Alternative therapies for CUD, including yoga, reiki, and hypnosis, were not well-known. In addition, only 3% of participants tried medications. Available off-label medications were considered unsuitable due to perceived factors like dependency, side effects, or concerns about being labeled a drug seeker.

Mobile applications were considered convenient but also thought to be impersonal and boring to use. Medical devices were mostly unheard of, and those individuals who were aware of them thought they were expensive and ineffective.

Concerns over lack of confidentiality was another barrier that prevented users from seeking out different therapies. However, participants had fewer concerns about visiting HCPs for medical care compared with those with current CUD.

In summary, in France and Spain, all participants tried counseling, and this was also a common therapy option in Brazil. Medication was considered more frequently in France than elsewhere. On average, the therapies detailed above were used less frequently in the UK than in other countries. Twelve-step programs were most widely used in the US, had a lower uptake in Spain, and were common in Brazil along with mobile applications. Residential rehabilitation was considered an option by participants in the US, but cost was a barrier.

See **Supplementary Material 4; Table S1** for a detailed summary of insights from individuals in CUD remission regarding current therapies and barriers.

#### 3.2.3 Supporters

Residential rehabilitation was favored by supporters looking for a total environment change. However, residential rehabilitation was considered expensive with a perceived risk of relapse upon returning to everyday life (**Table 2**). Counseling was considered meaningful. Social stigma and concerns over confidentiality were viewed as barriers that prevented their loved ones from seeking out the different therapies.

### 3.3 Insights from participants regarding future therapies

#### 3.3.1 Individuals with current CUD

Individuals with current CUD, when prompted to describe an “ideal” therapy, stated that they wanted a therapy that addressed cravings, withdrawal symptoms, and the “lows” of cocaine use (low mood, anxiety, and paranoia caused by the effect of cocaine wearing off) without developing a new addiction (**Table 3**). Access to these new therapies should be through HCPs but, again, users mentioned hesitancy about approaching HCPs.

**Table 3:** Insights from participants regarding future therapies, including an ideal tool to measure treatment efficacy.

Individuals with current CUD	Individuals in CUD remission	Supporters
•	•	•
Detailed insights		
• Oral treatment preferred – Stops/reduces cravings and targets withdrawal symptoms – Prevents cocaine from having an effect – Concerns about potential unintended consequences of treatment, such as overdose – Hesitance in accessing treatment via an HCP due to embarrassment and concerns about being labeled as a “drug seeker” – Some users would be comfortable accessing treatment through a third party such as a community clinic	• Holistic treatment preferred – Stops/reduces cravings and targets withdrawal symptoms as they are the key drivers of continued cocaine use – Helps individuals overcome their psychological issues – Needs to be a multifaceted approach to fulfill the many and varying needs of every individual with CUD – Treatment should be suggested by the professionals they see, e.g., therapist, but knowledge should also be shared through word of mouth – Treatment tailored to individual needs ■ Includes previous trauma and provides a safe supportive space ■ A remission/wellness plan that involves practical living and support arrangements to attain short- and long-term goals ■ Includes supporters when needed – Cost of treatment may act as a barrier if not reimbursable or covered by insurance	• Quick and convenient treatment preferred (preferably oral) – Stops/reduces cravings and targets withdrawal symptoms; there is the belief that these are the key barriers that prevent users from overcoming CUD and that cause the failure of previous therapies – Supporters are more likely than patients to feel that ideal treatment should also be convenient and quick to ensure patient compliance and treatment success • Intensive treatment with ongoing support – The treatment approach should be customized per an individual’s needs and be flexible to maximize the chances of ongoing engagement – Any treatment given must be intensive—either an inpatient treatment program or intense outpatient support—as supporters have watched their loved ones attempt to recover and fail many times – For this reason, many supporters are open to a medication approach, as they believe this may be the only thing that will work – Should include therapy for emotional and psychological support, such as counseling, a sober coach, spiritual advisers, a life coach, and character-building and goal-setting techniques • Practical support on issues such as housing, transport, jobs, money management and monitoring (to discourage relapse), nutrition, and sleep hygiene
• Potential for new mobile applications, CBT, peer support, etc.	• Oral treatment – Mixed reactions – Some are excited about any development in this area, particularly something that is simple and convenient – Can be similar to methadone (well-known across participants) – However, others are cautious of an easy fix, which may be like sticking a bandage over their problems – If treatment is too easy, it may result in an endless cycle of remission and relapse – There are concerns about contraindications and addiction to new treatment	• Oral treatment – Widely positive about an oral treatment – Medication is mentioned spontaneously as a desired treatment approach when discussing ideal treatment – Tend to feel medication is an unmet need for CUD, as they have seen their loved one with CUD fail (sometimes many times) with therapies – Oral treatment would be convenient and quick, so perhaps a higher chance of success even if patient motivation is challenging – A majority of supporters feel the individual with CUD would be open to medication ■ One supporter had discussed medication with the current user under their care, and they had said they would be open to it if it did not make them drowsy or spaced out
	• Realistic tools focused on the following should be used by HCPs – Most feel that efficacy can be assessed by clinical interviews and questionnaires (many have experience of this) – However, most assume that there is a risk that patients would lie about their cocaine use, so they also recommend: ■ Looking for “giveaways” e.g., non-verbal signs, lack of withdrawal symptoms ■ Verifying with other professionals or family support in contact with the patient ■ Building trust so that the patient can be honest ■ Regular urine/blood tests ■ Accessing physical appearance and signs, e.g., pupil dilation, weight gain ■ Encouraging patients to keep log of moods and cravings	• New treatment would be considered effective if the individual seemed like their old self again
	• Effective treatment would mean: – Feeling free from dependency – Improvement in physical, mental, and emotional health	

CBT, cognitive behavioral therapy; CUD, cocaine use disorder; HCP, healthcare professional.

#### 3.3.2 Individuals in CUD remission

**Figure 2:**
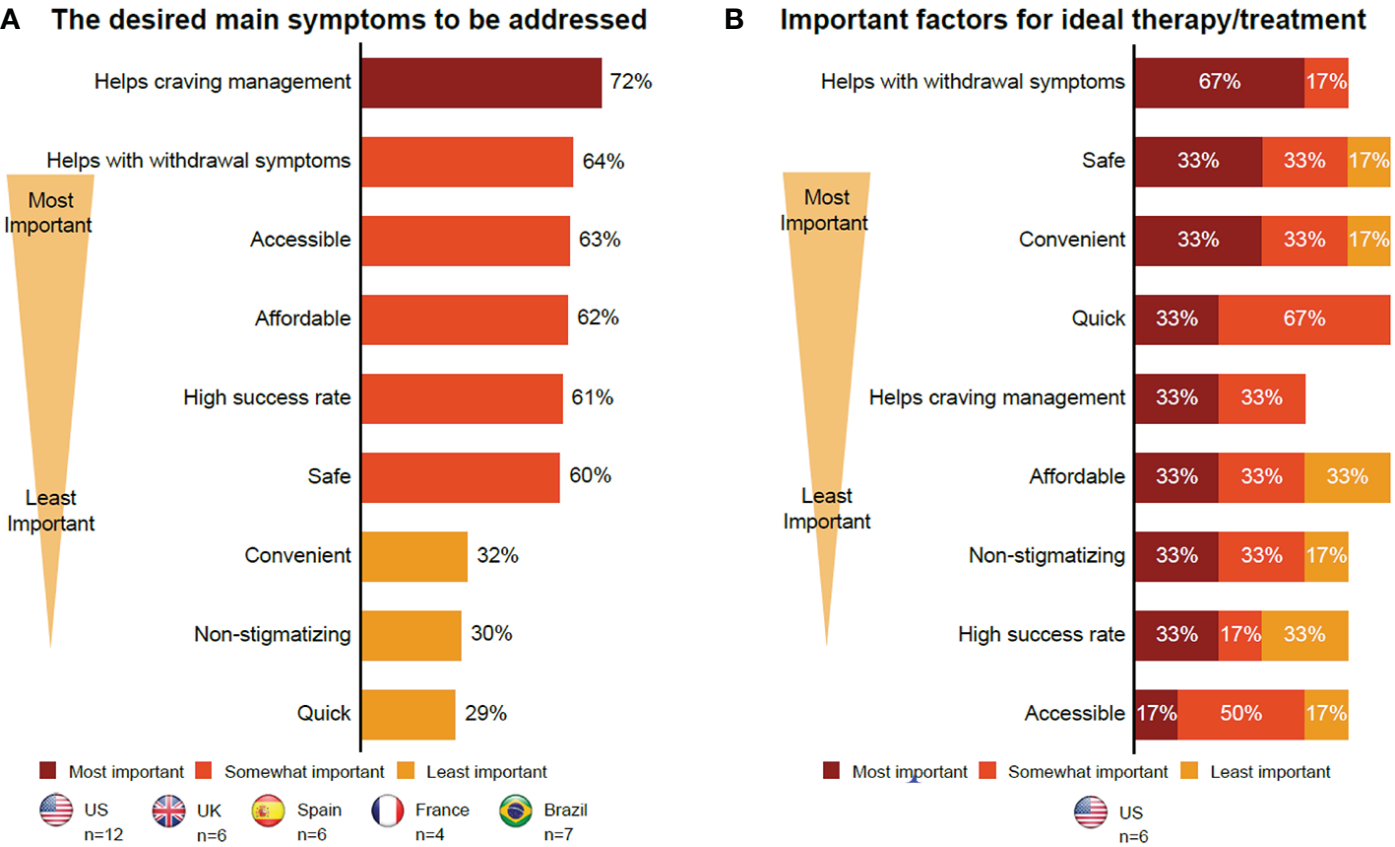
Insights into future therapies. **(A)** Individuals in CUD remission. **(B)** Supporters. The format of the focus group with those with current CUD did not allow collection of certain data. Therefore, this group is not included here. CUD, cocaine use disorder; UK, United Kingdom; US, United States.

Individuals in CUD remission had similar views on future therapies to those with current CUD, including the importance of new therapies stopping or reducing cravings (72% of participants) and withdrawal symptoms (64% of participants) (**Table 3**; **Figure 2**). Individuals in CUD remission were more interested in holistic treatment options that were tailored to individual needs. They felt that if a pharmacological treatment existed, it should exist alongside a support system that would promote physical and mental health, improve one’s quality of life (QoL), and everyday relationships. To determine how individuals are doing on a new therapy, they recommended realistic HCP tools that combined clinical interviews and questionnaires with parameters such as assessment on physical appearance, patient logs on moods and cravings, and verifications with family and other professionals in contact with the patient.

#### 3.3.3 Supporters

Supporters wanted therapies, especially pharmacological therapies, that would stop or reduce cravings (33% of participants) and withdrawal symptoms (67% of participants). They also wanted long-term intensive therapies that were holistic and affordable, and included mental support for users and care for supporters (**Table 3**; **Figure 2**). They also noted that these therapies should be convenient and quick to ensure compliance and long-term abstinence (**Figure 2**), with an emphasis on therapies that can improve QoL. Location, treatment flexibility, and willingness to undergo treatment were the barriers cited by the supporters. The cost of therapy was a potential barrier for supporters, if not reimbursable or covered by insurance (**Supplementary Material 5; Table S2**). Lastly, all participants had mixed views on pharmacological therapies due to concerns about safety, dependence, and an overall concern with pharmacological therapies being “easy fixes” that would cater more to the symptoms than the root causes of their CUD. In their opinion, this could lead to relapse.

### 3.4 Insights from participants regarding future clinical trials on CUD

Most participants across the three groups were interested in clinical trials (**Table 4**), but expressed some specific concerns, as outlined below.

**Table 4:** Insights from participants regarding potential clinical trials on CUD.

Individuals with current CUD			Individuals in CUD remission			Supporters		
Benefits	Barriers	Suggestions	Benefits	Barriers	Suggestions	Benefits	Barriers	Suggestions
• Anonymity provided in clinical trials • Potential “free” access to an innovative treatment that works • Helping others and scientific advancement • Want to see CUD being taken more seriously • Some think they might be paid to participate • Participants would be interested in learning more about clinical trials	• Would want to know the side effects of the medications required to be taken in the trial • Confidentiality very frequently mentioned—would like to be registered by number and not by name • Not comfortable taking treatment on camera • Visiting the trial center in person would be more straightforward/confidential	• Guarantee of anonymity • The doctor should check: – If the patient is still using cocaine – If the patient has used cocaine that day – If the patient has stopped using but used a few days prior – How long the patient has been using the drug – Levels of drugs in the patient’s system	• Helping others • and scientific advancement; some expressed particular interest in brain function; what works for different people; combination therapies • Want to see cocaine misuse being taken more seriously • Could help with additional tools to “stay sober” • Some think they might be paid to participate	• Some feel they would not be eligible as they no longer use cocaine • Concerns that taking part could be a trigger for use/relapse • Some were against medication in general • Concerns about side effects • Concerns about the hassle factor, e.g., duration • No real personal benefits perceived • Concerns about anonymity, and having to explain to others what they are doing • Not comfortable being filmed as part of the trial • Disappointment if they receive a placebo • Dislike of pharmaceutical companies	• Guarantee of anonymity • Clear explanation of purpose and process; consent should be obtained • Empathetic HCPs who treat trial participants with respect • No stigma attached to participation • Transparency of results • Provision of psychological support • Pleasant, relaxing environment with drinks and snacks (if visiting a clinic) • Ideally a payment or compensation provided to participants	• Save lives of people with CUD • Hope that after multiple failed attempts, finally a treatment that might work • Greater understanding of addiction, and how it affects those with CUD • Development of effective medications and treatment plans • The individual will get intensive physical and psychological monitoring and treatment, which they may not be able to access otherwise	• Concerns that participation could be a trigger for use/relapse in the patient • Unsure about their loved one’s willingness to participate (privacy issues and/or reluctance in stopping cocaine use) • Some were against medication in general • Concerns about side effects • Concerns about the hassle factor, particularly the duration of therapy • Concern about anonymity; would need reassurance on confidentiality • Concern the patient may be uncomfortable with taking treatment on camera due to the anxiety, stigma, and fears around privacy • Concern about monitoring triggering “paranoia” in the patient • Disappointment if the patient were to receive a placebo • Comorbidities • Dislike of pharmaceutical companies	• Guarantee of anonymity • Take time to explain trial process and monitoring carefully • Ensure the same trial personnel see the patient each time • Ensure the patient is not locked up or does not feel trapped • Pleasant, relaxing environment with drinks and snacks (if visiting a clinic)

CUD, cocaine use disorder; HCP, healthcare professional.

#### 3.4.1 Individuals with current CUD

Access to free trial medication, an eagerness to advance scientific research on CUD, and assured anonymity were key motivators to participate in a clinical trial (**Table 4**). Clinical trials were perceived as an opportunity for CUD to be taken more seriously in the medical and scientific fields. However, concerns about the potential side effects of medication and their privacy while being in a clinical trial were cited. Specifically, when prompted about their thoughts on taking medication via a video recording for compliance purposes, most participants were not supportive of this approach as it was considered intrusive. Although privacy was a concern, participants noted that anonymity in a clinical trial could also be a good motivator as they could take a potential trial treatment for their CUD without it being documented in their medical records.

#### 3.4.2 Individuals in CUD remission

**Figure 3:**
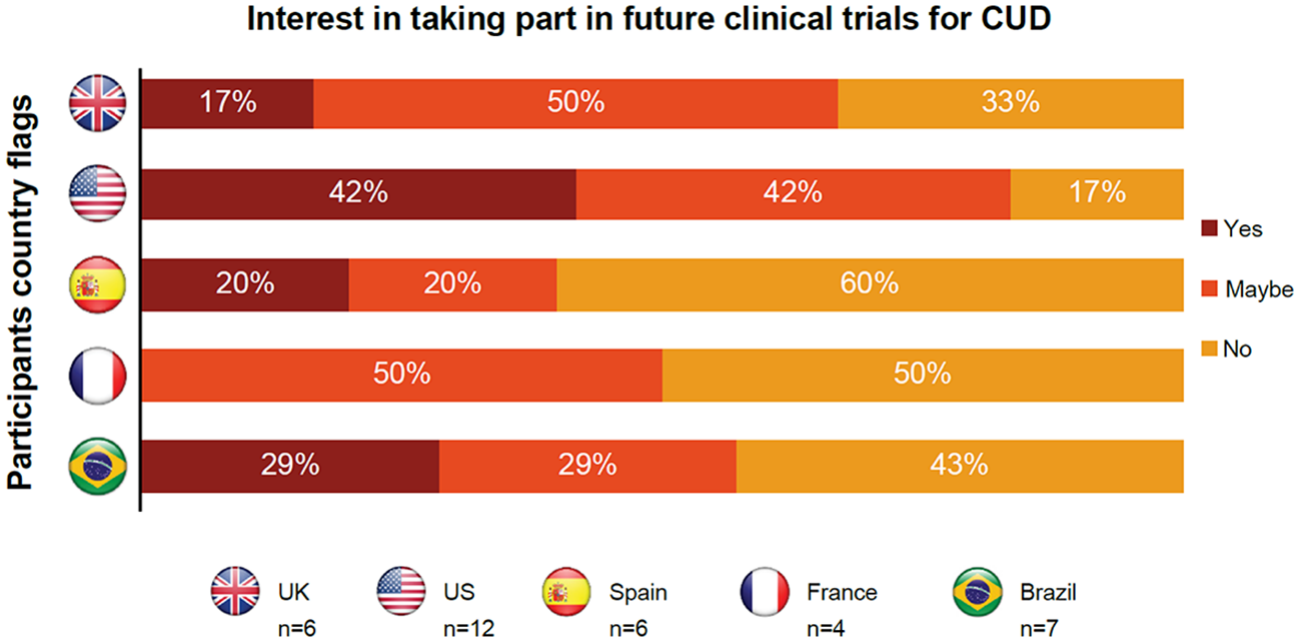
Insights from individuals in CUD remission regarding participating in future clinical trials for cocaine use disorder. CUD, cocaine use disorder; UK, United Kingdom; US, United States.

Individuals in remission had overall positive perceptions about participating in clinical trials but were concerned about their privacy and a lack of confidentiality (**Table 4**). They were also worried about receiving placebo instead of an active treatment. Participants from the US were most interested in clinical trial participation, with 42% of participants showing definite interest and 42% of participants showing possible interest (**Figure 3**).

#### 3.4.3 Supporters

Supporters thought that clinical trials could save lives and provide hope to loved ones who had multiple relapses (**Table 4**). However, they were skeptical about the willingness of their friends and relatives to participate in these trials due to their loved one’s potential lack of interest in stopping their cocaine use or concerns over confidentiality and privacy. Similar to those with current CUD, supporters were also uncomfortable about their loved ones taking treatment on camera.

Based on the insights collected regarding current and future therapies as well as regarding potential clinical trials, seven outcomes were suggested by participants for the success of a therapy. To achieve long-term abstinence, a blood/urine test was run in only individuals in CUD remission. To stop or reduce cravings, a patient log was maintained, and a clinical interview was conducted in individuals with current CUD and individuals in CUD remission. To feel free from dependency, a patient log was maintained, a clinical interview was conducted, and verifying with other professionals or supporters was done in individuals in CUD remission as well. To improve physical health/prevent long-term problems, a full physical exam and clinical tests were performed in individuals in CUD remission. To improve mental health, an interview with a trusted, empathetic person/read non-verbal signs was conducted in individuals with current CUD, individuals in CUD remission, and supporters. To improve QoL, including with work and relationships, an interview with a trusted, empathetic person/read non-verbal signs was conducted in individuals in CUD remission. To achieve all of the above, a realistic tool that combines clinical interviews and questionnaires with other parameters such as physical appearance, patient logs on moods and cravings, and verifications with family and other professionals in contact with the patient were performed in individuals in CUD remission.

## 4 Discussion

Studies on substance use and SUD have found individual counseling, CBT, and 12-step programs, among others, to be effective interventions (29–31). In the present study, counseling was the most popular therapy. Where it was available, CBT was popular among those looking for practical techniques to support their remission. Peer support groups and 12-step programs were of interest to older individuals in CUD remission (aged >49 years). Those with current CUD and some in remission found it challenging to connect with peers in a group setting and subsequently rejected the 12-step programs as outdated and religious in nature. Those with current CUD thought medication for comorbidities could be useful, but going to an HCP generated concerns about privacy, anonymity, stigma, and documented treatment. Both current and recovered individuals with CUD were concerned about off-label medications leading to dependence, or about being perceived or labeled as drug seekers. Residential rehabilitation was not frequently tried due to the high cost. Alternative medicine was not well-known as a CUD therapy. Medical devices and mobile applications were the least-used approaches for CUD therapy.

Costs of therapy, lack of anonymity, and social stigma were the frequently cited barriers to treatment for all groups of participants in this study, similar to other studies (32). The time required for therapy was a major barrier for those with current CUD, whereas location and flexibility of therapy were a concern for the supporters.

In the present study, those with current CUD were most interested in new therapies, and those in remission and supporters were more focused on other aspects such as psychosocial therapies. Although supporters were looking forward to fast-acting, effective oral therapies, those with current CUD or in remission were fearful about dependence or quick fixes where the root cause remained unaddressed. However, a common point of agreement among all participants was that a new therapy could stop or reduce cravings and withdrawal symptoms, and achieve long−term abstinence.

Despite the potential drawbacks identified for clinical trials, such as intrusivity of being filmed if that was part of the clinical trial compliance measures, the interventions and questions in clinical trials as a possible trigger for relapse and paranoia, side effects, and concerns over receiving a placebo, there was a general interest and desire to contribute to science. The anonymity and privacy afforded by a clinical trial setting were also viewed favorably.

Limitations of the study included a modest study sample. Owing to the limitations of the format of the focus group, some questions varied between the OBB and the focus group. Because of cultural differences toward addiction and the willingness to talk about it, individuals with current CUD and their supporters were only from the US, which may have led to bias in the results. Consistent with qualitative research, the patient population was not balanced as in a standard clinical trial. Moreover, some participants (6% of the individuals in CUD remission) had no diagnosis consistent with CUD, although they did have at least five criteria of SUD per DSM-5 criteria.

## 5 Conclusion

Experiences with therapy varied among participants, and several attempts were often needed to stop or reduce cocaine use. Satisfaction with the available treatment options was low, with concerns about efficacy, therapies not being tailored to cocaine use, and cost to the patient. A holistic treatment—that is, pharmacological treatment with personalized therapy that helps reduce cravings and is affordable—was considered as ideal. Interest in a potential new oral treatment was mixed, with some concerns around dependence or misuse. Patient information and support tailored to stopping cocaine use would be beneficial, particularly if it is adaptable to the unique psychosocial needs of the individuals with current CUD or in CUD remission.

Long-term abstinence may be the main goal for a clinical trial; however, based on the perspectives of individuals with current or past CUD and supporters, other outcomes such as reduced cravings, improvement in physical and mental health, improvement in QoL, and prevention of long-term problems were also viewed as important considerations for potential clinical trials. Therapy outcomes need to include patient-led evaluation and objectives to realistically assess if the patient is still using cocaine.

Enthusiasm about clinical trials was high among the participants due to the anticipation of improved treatment options specific to CUD. However, those with current CUD reported many barriers to participation, particularly paranoia, which could be worsened if the trial required treatment compliance to be documented by video recording. This study suggests that participants require efforts to be made on building trust and ensuring the privacy of their personal information. Furthermore, although many legal and ethical safeguards exist to ensure anonymity and privacy of a clinical trial participant’s data, this study made it clear that special care must be taken to explain to participants in CUD trials about their privacy rights and provide additional plain language educational materials regarding their privacy rights outside of the informed consent form. Participants had many concerns about their privacy and documentation of a potential CUD prescription drug in their medical record. As medical record privacy is a major barrier to individuals seeking professional help, it is important to identify ways to create an environment of assured privacy and anonymity once a medication is available on the market (e.g., coding the medication in the record).

With a growing interest in patient-informed clinical trial designs, this study highlights the unmet needs of individuals with current, or in remission from, CUD and their supporters. There is an urgent need to continue investing in research for new CUD therapies, given the scope of the problem and the soaring rates of cocaine use globally.

## Data availability statement

The original contributions presented in the study are included in the article/**Supplementary Material**. Further inquiries can be directed to the corresponding author.

## Ethics statement

This study was conducted in accordance with EphMRA guidelines which state that ethics committee approval is not required if study met the criteria of market research. Therefore, Institutional Review Board approval was not sought for this market research study. All procedures involving human participants were performed in accordance with the declaration of Helsinki and guidelines for Good Epidemiology Practice. Standard procedures were adhered to for the protection of participants’ rights, including written informed consent, data privacy, anonymity, and the right to withdrawal, as well as thorough assessment of the qualifications of the vendor was undertaken. The participants provided their written informed consent to participate in this study.

## Author contributions

SM: Conceptualization, methodology, visualization, project administration, funding acquisition; writing the original draft, reviewing and editing the subsequent drafts. DL: Conceptualization and writing the original draft; visualization, supervision, funding and acquisition; reviewing and editing the subsequent drafts. BG-M: Conceptualization, methodology, visualization, supervision, funding and acquisition; reviewing and editing the subsequent drafts. BK: Visualization and writing the original draft; reviewing and editing the subsequent drafts. VSM: Formal analysis and data curation. PB: Conceptualization, methodology, writing the original draft; reviewing and editing the subsequent drafts. SD: Writing the original draft and reviewing and editing the subsequent drafts. KW: Conceptualization, methodology, validation, visualization, project administration; writing the original draft; reviewing and editing the subsequent drafts. All authors contributed to the article and approved the submitted version.
