# Supplementary material for: Patient perspectives on current and potential therapies and clinical trial approaches for cocaine use disorder
Source: Front Psychiatry. 2024 Feb 29;15:1230699. doi: 10.3389/fpsyt.2024.1230699 (PMC10937549; doi:10.3389/fpsyt.2024.1230699)
Supplement: Supplementary file 2 [file DataSheet_2.docx]

# Table S1. Insights from individuals in CUD remission on current therapies/community mutual help groups used by CUD patients

|  | **Knowledge** | **Access** | **Pros** | **Cons** | **Favored by** | **Type of response received** |
| --- | --- | --- | --- | --- | --- | --- |
| **Counseling** | - Widely known about and used - ‘Logical’ place to start for addiction and mental health issues | - A few received counseling for free or with their insurance, but most said it was only available to them privately at an additional cost | - Judgment-free social interaction with a neutral professional - Addresses underlying triggers/ trauma - Overall feeling is that it can work - A good fit with the therapist is necessary - Relieves family/friends of the burden of talking through problems | - Painful process - Time consuming - Requires motivation - Stigma felt for some - Very generic and not specific to CUD - Cost or a lack of health insurance coverage was a barrier for patients accessing therapy | - Wide relevance | - *“It gave me the tools to be able to sort of manage some parts of my life. It gave me the chance to express myself.” Spain* |
| **Lifestyle changes** | - Lifestyle changes generally taken to mean reducing consumption or total abstinence through changed habits | - There are no cost-related barriers, but a high level of motivation is required in order to abandon and change old patterns of doing things | - Can ‘break cycle’/unhelpful relationships - Appeals to those who are more self-reliant than others - Willpower and abstinence are often considered the foundations of remission | - Difficult; requires discipline, willpower, and motivation - Desire to abandon personal identity and friends - Can be a drastic change - Hard to achieve alone | - Appeals most to individuals in CUD remission who are more likely to prefer self-reliance and resent intrusion from ‘professionals’ | - *“That enables you to get rid of these patterns, it enables you to go to meet new groups and networks based on remission.” France* |
| **12-step program** | - Widely known about, often referred to as Narcotics Anonymous | - Accessible and free for most - Can be referred as part of a compulsory program by law | - Belief it can work for some people - Community, solidarity - Structured way to make a fresh start | - A group format is not appropriate for everyone - Feels old-fashioned, cliched or semi-religious to some - Stigma – for ‘addicts’ - It requires a long-term commitment | - Appeals most to older individuals in CUD remission, some of whom had become volunteers or had lifelong friends from 12-step programs | - *“Puts you in touch with other people who understand you.... it is good to share my progress.” UK* |
| **CBT** | - Awareness of CBT was good, but not everyone was clear on the specifics - Most, but not all, recognized CBT as being different to counseling due to the focus on changing thinking patterns | - Available through the National Health Service in the UK - Some mention of free but compulsory access via law enforcement/probation - Privately accessed treatment can be expensive in other markets | - Teaches a new logical way of thinking and coping - Focused on practicalities and behavior - Believe that it can work for some people | - Can take a long time to start working - Some doubt whether it addresses underlying issues - Can feel cold/mechanical to some | NA | - *“A more concise insight into the brains input of addiction and the circles that we create.” UK* |
| **Individual/family therapy** | - Most do not differentiate between ‘therapy’ and ‘counseling’ except for the element of family involvement | - Often adjutant/referred via another therapy | - Promotes open dialog with family members - Makes it easier for family members to understand - Thought to aid the healing process and underlying drivers | - Some do not feel they have good enough relationships with their family to ask for their participation, and family members may have scheduling issues - Can be painful/difficult to confront - Seen as for more ‘extreme’ problems/addictions by some - Younger people may do it for their parents | - Appeals most to individuals in CUD remission aiming to mend broken relationships | - *“Remission is a team effort, and it helps all those impacted to understand the remission path and the part they played in the addiction.” UK* |
| **Residential rehabilitation** | - Rehabilitation widely known about, though the quality is felt to vary according to whether they are state-supported compulsory programs or ‘celebrity’ retreats | - The cost can be a significant barrier, especially for more ‘desirable’ rehabilitation brands/locations | - Protected, safe environment with no illicit drugs and with time to detox - Total immersion to break negative habits and routines - Holistic approach with lots of different tools available during the stay | - Not a ‘real’ environment - Risk of relapse on return - Long stays not practical, especially for parents - Feels like incarceration to some | - Appeals most to younger individuals in CUD remission, and to individuals in CUD remission with the resources to pay | - *“Inpatient provides the opportunity to have a separation from toxic environment and focus solely on getting into a remission process.” US* |
| **Peer support** | - Peer support groups are typically seen as similar to 12-step programs in terms of the group element | - Easily accessible and free | - Creates a sense of community, democratic, peer-led - Provides support and accountability - Support is available outside normal/conventional hours | - Potential negative impact from peers - Group therapy is not preferred by some people - Skepticism about efficacy | - Appeals most to older individuals in CUD remission, some of whom get involved in the recovered cocaine user ‘community’ | - *“Sharing experiences, identifying with others, being inspired by others” Spain* |
| **Alternative medicine** | - Many people lack knowledge/ interest about the potential of alternative medicine - A few had tried things like yoga, reiki or hypnosis | - Typically, only available privately and therefore can be costly - Some alternative therapies are available within residential rehabilitation programs | - Helps with relaxation - Reduces anxiety - Wider lifestyle and mental health benefits | - Skepticism about efficacy, especially from those who had already tried alternative medicines for ‘lesser’ addictions (e.g., for smoking cessation) - Practitioners are typically not specialized in addiction | - Niche appeal | - *“Yoga is great for helping with anxiety” UK* |
| **Medications** | - Good awareness that medication can be used for some addictions (e.g., methadone, for opioid addiction) or for comorbidities (Ritalin) but is not yet available for cocaine | - A minority had accessed medication for other addictions - Usually covered by healthcare insurance/public health services | - Scientifically backed remission - A helping hand - Can be used when other methods have failed or in conjunction with other methods | - Still dependent on a product, which some feel is not ‘true’ remission - Concern about dependency and side effects - Concern about being seen as ‘drug seekers’ by HCPs (individuals with current CUD) | NA | - *“Scientifically backed proven remission treatments, typically provided by a source that allows for further connection to remission resources.” Brazil* |
| **Mobile applications** | - Limited awareness of anything specific for cocaine | - Convenient and low or no cost | - Private and accessible - Structured support that can be tailored - Ability to get information from peers | - Can feel impersonal - Can be boring in comparison to face to face - Considered unlikely to work in isolation | NA | - *“Mobile application gives you the ability to connect anytime” Brazil* |
| **Medical devices** | - Low awareness of medical devices to help with cocaine use | - Expensive, which is likely to be a barrier for access | - None found in this sample | - Expensive and ineffective | NA | NA |

Abbreviations: CBT, cognitive behavioral therapy; CUD, cocaine use disorder; HCP, healthcare professional; NA, not applicable; UK, United Kingdom; US, United States

# Table S2. Barriers to future treatments for supporters

| **Barriers** | **Comments** |
| --- | --- |
| - Cost of treatment may act as a barrier if not reimbursable or covered by insurance - Location and flexibility around other commitments (e.g., work and children) are also important - The main barrier to access is willingness of the person with current CUD or in remission from CUD themselves to undergo therapy - Residential programs turn away CUD patients and focus on users of alcohol, pills, and heroin | - *“A lot of treatment facilities say you don't have withdrawal from cocaine and won't take patients. They mainly focus on alcohol, heroin, pills” Caregiver, US* - *“My sister does not like meetings, she does not want therapy, she just wants to pretend everything is fine.” Caregiver, US* |

Abbreviations: CUD, cocaine use disorder; US, United States
